# Variations in origin of middle hepatic artery, a CT angiographic study

**DOI:** 10.6026/973206300214260

**Published:** 2025-11-15

**Authors:** Stuti Tandon, Garima Sehgal, Manjari Lohani, Raveendra Singh Rajpoot, Deepanshu Shukla, Rehan Ahmed, Saket Kumar

**Affiliations:** 1 Department of Anatomy, Integral Institute of Medical Sciences and Research, Integral University, Lucknow, India; 2 Department of Anatomy, King George's Medical University, Lucknow, India; 3 Department of Anatomy, Prasad Institute of Medical Sciences, Lucknow, India; 4 Department of Radiodiagnosis, Dr. K. N. S. Memorial Institute of Medical Sciences, Barabanki, Uttar Pradesh, India; 5 Department of Anatomy, T. S. Misra Medical College and Hospital, Lucknow

**Keywords:** HAS variations, CT angiography, left hepatic artery, right hepatic artery, middle hepatic artery, hepatic artery proper

## Abstract

A detailed understanding of hepatic arterial and portal venous anatomy is essential for liver-centric therapies and surgical procedures, especially transplantation. This cross-sectional CT angiographic study was conducted on 50 subjects (12-75 years) at King George's Medical University, Lucknow. The proper hepatic artery divided into Right Hepatic Artery (RHA) and Left Hepatic Artery (LHA) in 93.1% of cases, while trifurcation was rare (4%); overall, 42% showed arterial variations. The middle hepatic artery (MHA) most commonly arose from the LHA (44.7%) or RHA (31.9%), with dual arterial supply to segment IV in 6% of cases. Identifying these variations through CT angiography is crucial for preoperative planning to optimize surgical outcomes and reduce complications.

## Background:

Variations in the vascular anatomy of the liver have also been classified and proposed by Hiatt in 1994. He proposed modification to Michels classification and described six types of arterial anatomy [1]. According to the Couinaud's classification, the liver is divided into eight segments which are functionally independent and have separate branch of portal vein, hepatic artery and bile duct. On entering the liver parenchyma, the right hepatic artery divides into an anterior branch supplying segments V and VIII, and a posterior branch supplying segments VI and VII. The anterior branch often supplies a twig to segment I and gallbladder. The left hepatic artery runs vertically towards the umbilical fissure, divides into medial and lateral segmental branches supplying segments I, II and III. Segment IV lies medial to the falciform ligament and is divided into IV a (superior) and IV b (inferior). Unlike other segments of the liver, arterial supply of segment IV has remained a confusing entity. The MHA is usually the artery to the segment IV of the liver [2]. It usually arises from the left hepatic artery at the hilum to supply the segments IV a and IV b. Identification and pattern of supply via Middle Hepatic Artery is important to know as it supplies the segment IV of the liver and damage to this artery is associated with various complications [3, 4]. Origin of MHA is subject to variation; MHA has been reported to originate variably as a branch from RHA, from trifurcation of PHA, from LHA with replaced RHA or from RHA with replaced LHA [5]. Usually, MHA displays an intrahepatic origin and course but it may arise extrahepatically when the bifurcation of PHA is low and traverse the Calot's triangle putting it at risk during cholecystectomy. It may give rise to hepatic falciform artery [6]. Preoperative imaging is utilized to properly delineate and understand the hepatic arterial system and to appreciate the anatomy and relations with other organs in all dimensions [7, 8, 9-10]. With the advent of multi-detector CT (computed tomography) it becomes necessary to redefine and study the hepatic artery anatomy; thin-section dynamic CT provides more detailed assessment of hepatic vasculature in comparison to angiography used previously for preoperative evaluation as it is more convenient for patients (obviating analgesia and periprocedural nursing care) and without the accompanying morbidity and mortality associated with catheter angiography [11, 12-13]. Incidence of variation in Hepatic Arterial System (HAS) ranges from 20% to 50%. Such variability in reported prevalence among different populations uncovers the uncertainty of scientific evidence on this issue, questioning the universal and indiscriminate applicability of the information, particularly when populations with different ethnicities are considered [14, 15, 16-17]. Knowledge of anatomic variations in hepatic vasculature is of great importance in hepatic and pancreatic surgeries and is also important with regard to laparoscopic surgery, radiological procedures, and the treatment of penetrating injuries involving the peri-hepatic area [14]. Angiographic demonstration of normal variants in HAS is especially important while planning whole and split liver transplantation and also, when considering presurgical planning, catheterization, and trans-arterial chemoembolization (TRACE) [18]. Thus, studying the anatomical pattern of such arteries on Indian populations is desirable and pertinent. Therefore, it is of interest to observe CT images belonging to patients subjected to abdominal contrast-enhanced multi-detector CT scans and aimed at analyzing the prevalence of anatomical variations of hepatic arterial system with special focus to MHA anatomy and variations.

## Materials and Methods:

The present cross-sectional, observational study examined CT data belonging to 50 subjects (including both males and females) in the age group between 12 years to 75 years. The study was conducted in the Departments of Anatomy and Radiodiagnosis, King George's Medical University, U.P., Lucknow during September 2015 to August 2016. This study was approved by the Ethical Committee of the University as per the Helsinki Declaration of 1975, as revised in 2000. CT Angiography was performed on a 64 slice CT scanner. CT data of all persons, male or female of all ages undergoing abdominal CT for any indication was collected retrospectively and all CT images were subjected to analysis. Wherever, the data revealed findings suggestive of aorto-arteritis, collagen vascular disorder, abdominal malignancy, any pathology in the region of interest that distorted the vascular anatomy or when it was obtained from patient with history of previous abdominal surgery it was excluded from study. Finally, after due consideration to all exclusion criteria, CT data belonging to 50 subjects was included in the study for making observations on hepatic arterial system anatomy. The dynamic 64 slice contrast CT angiography images of the Triple Phase of Abdomen were evaluated and arterial anatomy was observed. PHA anatomy was observed regarding presence or absence of artery as well as its branching pattern. When PHA was the continuation of CHA, gave off gastroduodenal artery and finally terminated into its branches it was considered as present; whereas any deviation from this pattern (early branching of left/right hepatic artery before the origin of GDA (gastro duodenal artery); RHA / LHA being replaced or in combination of replaced & accessory hepatic arteries) was categorized as absent PHA. The origin of segment IV artery was also observed and documented. The data obtained was subjected to statistical analysis using SPSS (Statistical Package for Social Sciences) Version 15.0 statistical Analysis Software. The values were represented in number (%) and Mean ± SD.

## Results:

Out of 50 cases, 29 (58.0%) were males and 21 (42.0%) were females, distribution of cases is shown in Table 1 (see PDF); Figure 1 (see PDF). PHA was seen to branch off into variable number of branches. PHA dividing into two branches (RHA & LHA) was seen in majority (93.10%) (Figure 3 - see PDF) as compared to its division into three branches; where the three branches of PHA included RHA, LHA & MHA (Figure 4 - see PDF). A higher proportion of female subjects (61.90%) displayed two branches of PHA (LHA, RHA) as compared to a smaller proportion (9.52%) with three branches (LHA, MHA, RHA). In 48.28% of males PHA divided into two branches LHA & RHA; three branches pattern was not found in any of the male subjects. Difference in branches of PHA in male and female subjects was not found to be statistically significant (p=0.094); (Table 2 - see PDF). Early branching of left/right hepatic artery before the origin of GDA; RHA / LHA being replaced or in combination of replaced & accessory hepatic arteries) was categorized as absent PHA. Artery to hepatic segment IV was seen present in all subjects. Single segment IV artery was observed in 47 subjects (94%) whereas two segment IV arteries were observed in 3 subjects (6%). Source artery of Segment IV artery was traced and it was observed that single segment IV artery was derived from LHA, RHA, PHA or CHA in 47 subjects; origin of MHA was most commonly from LHA in 21 subjects (44.68%) (Figure 6 - see PDF), followed by RHA in 15 (31.91%) (Figure 8 - see PDF), from CHA in 9 (19.15%) (Figure 5 - see PDF) & from PHA in 2 (4.26%) (Figure 4 - see PDF). Amongst 3 subjects with dual supply for segment IV; the upper and lower segment IV arteries originated from LHA and RHA respectively in 2 subjects (Figure 7 - see PDF) whereas in 1 case the artery to lower segment IV and upper segment IV were branches of LHA and Acc. LHA respectively. Difference in origin of segmental supply to hepatic segment IV/MHA between males and females was not found to be statistically significant (p=0.591) (Table 3 - see PDF) (Figure 2 - see PDF).

## Discussion:

There has been increase in surgical interventions and organ transplant procedures these days, resulting in an increase in the relevance of discussion about variations in the hepatic arterial pattern. There have been many publications about extrahepatic arterial anatomy [2]. It is very crucial to have a detailed knowledge of extrahepatic arterial anatomy if we wish to avoid damage of donor, recipient and the graft too. Though most of the studies done so far have used conventional angiography [19], the present study was conducted to identify the various patterns of branching of the proper hepatic artery and various patterns in origin of artery supplying segment IV by using MDCT (multi detector computed tomography) angiography. Proper hepatic artery is described as the continuation of the common hepatic artery (CHA) after the branching off of the gastroduodenal artery (GDA). PHA as described above was found to be present in 58% subjects in the present study. In the remaining 42% subjects; early branching of left/right hepatic artery before the origin of GDA; RHA / LHA being replaced or in combination of replaced & accessory hepatic arteries, the PHA was considered as absent. Accordingly, on the basis of the mentioned criteria in labelling absent PHA, observations among the study subjects revealed a high prevalence of absent PHA (42%), thereby indicating that a high proportion of study subjects revealed variation in anatomy and early branching of right and left hepatic arteries. The middle hepatic artery was found to be originating from both right and left hepatic artery with equal frequency by Michels & Healey [2, 20]. Many other studies claimed middle hepatic artery originating from left hepatic artery with higher frequency (54%-61.5%). The middle hepatic artery was reported to be originating from Left hepatic artery in 54% and from Right hepatic artery in 34% of cases by Suzuki, this study also found the bifurcation of proper hepatic artery in 8% and found it to be coming as direct branch of common hepatic artery in 4% of cases [21]. Onishi *et al.* reported middle hepatic artery to be originating from left hepatic artery in 61.5% and from right hepatic artery in 27.5% [22]. Ozsoy *et al.* (2011) recognized early branching of right and left hepatic arteries in 33 out of 496 potential liver donors who underwent donor hepatectomy surgery [18]. Early branching of LHA was also seen by Sureka *et al.* (2013) in 0.5% cases. In such cases left and right hepatic arteries originate from CHA [23]. When RHA originates from the common hepatic artery (CHA) or when the LHA arises before GDA, the perfusion to the stomach and duodenum may decrease on clamping of the CHA. Presence of such variations would affect the surgical outcome and hence prior knowledge may help to modify the surgical process for a favourable outcome.

In 54% cases, PHA divided into 2 branches, LHA and RHA; the branching seen classically and described in textbooks whereas, in 4% cases PHA divided into 3 branches i.e. LHA, MHA and RHA. When GDA, LHA and RHA all arise together from CHA (trifurcation), arterial ligation during surgery needs special consideration [24]. Arterial supply to segment 4 of liver lacks consensus in segmental liver anatomy [25]. It is very important to be certain of the origin of blood supply to the segment 4 of liver before embarking upon transplant surgery. In order to get clear knowledge of hepatic arterial supply including the very fine calibre vessels to segment 4, MDCT is of great help [26]. Arterial supply to segment 4 from RHA has been reported to be present in 62.5% of cases by Kamel *et al.* and in 9% of cases by Erbay *et al.* using MDCT [27, 28]. In a similar study the origin of segment 4 supply has been reported to be originating from RHA in 41.33%, from LHA in 27.83% and from CHA in 4.5% cases by Sureka *et al.* in about 26.3% of cases the origin of arterial supply of segment 4 was not clear. MHA was not found to be arising from accessory hepatic artery [23]. Coincidently whenever the replaced LHA arises from LGA, MHA was found to the originating from RHA, and whenever a replaced RHA was arising from SMA or celiac axis, the MHA arose from LHA. In our study it was observed that MHA originated from LHA in 44.68%, from RHA in 31.91%, from CHA in 19.15% and from PHA in 4.26%. Three additional MHAs were also observed in three subjects who received two arteries for segment four. According to Wang *et al.* five types of MHA have been defined; Type I MHA (MHA arising from RHA); Type II MHA (MHA arising from LHA); Type III MHA (MHA arising from RHA along with a replaced LHA); Type IV MHA (MHA arising from LHA along with a replaced RHA) and Type V MHA (MHA arising from CHA, PHA and the right anterior hepatic artery) [29]. Ghosh in his cadaver-based study found that in presence of accessory LHA the MHA arose as a subbranch of RHA in 15% of cases [30].

Jin *et al.* described that during early embryonic development of liver it has three lobes which are divided into four sectors: the lateral sector which is comprised of segment 2 is supplied by embryonic LHA, the medial and anterior sectors which are comprised of segments 3, 4, 5, and 8 are supplied by embryonic MHA which in turn is coming from common hepatic artery, and the posterior sector comprising segments 6 and 7 is supplied by embryonic RHA. Persistence of this embryonic arterial pattern gives rise to variations in hepatic arterial supply [25]. In adults, the left lobe of the liver is supplied by the LHA and the right lobe of the liver is supplied by the RHA. So, there is a codominant supply of liver by LHA and RHA. The term middle hepatic artery has been used in most of the studies for A4 (artery to segment 4) and have shown it to be arising frequently from LHA (54%-61.5%). When A4 arises from RHA, it is said to the dominant artery. Studies using MDCT, done on living donor transplantation recipients have shown artery to segment 4 to be originating from RHA in 55% to 62.5% of the cases [25, 31, 32]. In a meta-analysis MHA has been found to be giving origin to the right gastric artery in about 1.31% of study subjects, further adding to the intricate relations it has with the different arteries of the region [33].

In our study segment IV artery arises from LHA in 47.17 % (25 arteries out of 53) of cases (LHA was the dominant artery), from RHA in 32.08 % (17 out of 53) of cases, from CHA in 16.98 % cases (nine out of 53), and from PHA in 3.77 % of cases (2 out of 53). In one case of accessory LHA, upper hepatic segment IV was supplied by accessory LHA and lower segment IV was supplied by LHA. In two cases A4 originated from both LHA and RHA in which upper segment IV was supplied by LHA and lower segment IV was supplied by RHA. A meta-analysis including 15 studies and almost 3,819 subjects has come up with the data which closely resonates with our study [34]. Information on the vascular origin and variations on segment 4 arterial supply is of great importance in transplantation surgeries. While undertaking right lobe transplantation, if the artery to segment 4 is found to be originating from RHA, the RHA should be divided distal to the origin to segment 4 arteries, to avoid the ischemic damage to the left lobe of the donor [35]. Knowledge of LHA variation is equally important during left lobe donation. Knowledge about HAS variations is helpful in guiding resection line during split liver resection or trisegment transplantation and while planning for liver partition along with portal vein ligation in staged hepatectomy for managing hepatic malignancies and metastasis surgically. Prior knowledge of existence of variations in MHA helps in avoiding ischemic damage and cholangiopathy in segment IV and also guides in division of the hepatic tissue on the basis of the arterial supply and the intrahepatic blood distribution to the segment IV.

## References

[1] Hiatt JR (1994). Ann Surg..

[2] Michels NA (1966). Am J Surg..

[3] Courtney M, Townsend Jr-MD (2012). Sabiston textbook of surgery International Edition..

[4] Holbert BL (1996). AJR Am J Roentgenol..

[5] Tajima T (2009). Acta Radiologica..

[6] Koops A (2004). Surg Radiol Anat..

[7] Douard R (2002). Transplantation..

[8] Catalano OA (2008). RadioGraphics..

[9] Winston CB (2007). AJR Am J Roentgenol..

[10] Nghiem HV (1999). Abdom Imaging.

[11] Takahashi S (2002). Radiology..

[12] Lee SS (2003). Radiology..

[13] Suzuki T (1971). Am J Surg..

[14] Song SY (2010). Radiology..

[15] Lezzi R (2008). Surg Radiol Anat..

[16] Sebben GA (2013). Rev Col Bras Cir..

[17] Liu CL (2006). Ann Surg..

[18] Ozsoy M (2011). ISRN Surg..

[19] Choi TW (2021). Radiol Cardiothorac Imaging..

[20] Healey JE-Jr (1953). J Int Coll Surg..

[21] Suzuki H (1982). Nihon Geka Hokan..

[22] Onishi H (2000). Hepatogastroenterology..

[23] Sureka B (2013). Indian J Radiol Imaging..

[24] Duran C (2009). J Comput Assist Tomogr..

[25] Jin GY (2008). Liver Transpl..

[26] Ugurel MS (2010). Br J Radiol..

[27] Kamel IR (2001). AJR Am J Roentgenol..

[28] Erbay N (2003). AJR Am J Roentgenol..

[29] Wang S (2010). Liver Transpl..

[30] Ghosh SK (2014). Anat Cell Biol..

[31] Ashok P (2022). JCDR..

[32] Mizumoto R, Suzuki H (1988). World J Surg..

[33] Lastoria DAA (2023). Surg Radiol Anat..

[34] Triantafyllou G (2025). Surg Radiol Anat..

[35] Yoshimura H (1986). Eur J Radiol..

